# ‘Going dark’ or under the radar? Challenges and opportunities for local authorities and dark kitchens in ensuring food safety

**DOI:** 10.1016/j.foodcont.2025.111179

**Published:** 2025-01-25

**Authors:** Zainab Laheri, Iain Ferris, Diogo Thimoteo da Cunha, Jan Mei Soon-Sinclair

**Affiliations:** 1 School of Health Sciences, Social Work and Sport, University of Central Lancashire, Preston, PR1 2HE UK; 2 School of Chemical Engineering, University of Birmingham, B15 9TT UK; 3 Universidade Estadual de Campinas, Faculdade de Ciências Aplicadas, Laboratório Multidisciplinar em Alimentos e Saúde, Limeira, Brazil

**Keywords:** delivery, food business operators, environmental health officers, online aggregators

## Abstract

Traditionally takeaway food outlets have relied on passing trade or it was included as part of a restaurant’s offering. Yet the surge in online applications for ordering food has challenged this operational model. The rise of so-called dark kitchens that have no physical customer-facing presence has revolutionised the takeaway food sector. This change comes with a unique set of challenges and opportunities for food safety inspections and implementations. This study aims to assess the challenges in identifying and regulating dark kitchens, and to identify potential interventions to increase food safety compliance in dark kitchens by working with local authorities and dark kitchen owners and tenants. A mixed-method study involving a cross-sectional survey (n=123) and two focus group discussions with 16 Environmental Health Officers (EHOs) and 16 semi-structured interviews with dark kitchen owners and tenants were conducted in England. Our study revealed multiple challenges faced by dark kitchen operators in managing food safety in shared spaces, food handling during delivery, high turnover of staff and delays in updating menu changes with online aggregators. The latter part of our study highlights the challenges encountered by EHOs in identifying and inspecting dark kitchens; including resource constraints, lack of dark kitchens’ visibility, multiple trading names, insufficient guidance from regulatory body, communication difficulties, difficult working conditions in some dark kitchens and problems identifying where responsibility lies. Based on the perspectives of EHOs and dark kitchens, practical recommendations to improve food safety standards of dark kitchens are provided. The study also highlights the important role the online aggregators play in supporting Local Authorities as they have the ability to monitor and ensure rigorous vetting of food businesses before onboarding the food business. This is the first empirical study to assess the challenges in identifying and regulating dark kitchens as well as to identify the challenges and opportunities for food safety implementation and inspection in dark kitchens.

## 1 Introduction

In recent years, the food industry has been revolutionised by the emergence of delivery only services called dark kitchens (Hakim et al., 2020). Dark kitchens (DKs) are food services that offer ready-to-eat meals for delivery or takeout through online platforms, including social media, mobile applications, restaurant websites or via phone/email. They may or may not have a storefront but do not offer a space for on-site dining (da Cunha et al., 2024; Nigro et al., 2022). The term ‘dark’ in dark kitchens refers to a lack of visible retail presence. However, there is a negative connotation associated with the term ‘dark’ (Hakim et al., 2020), thus they may also be referred to as ‘delivery-only kitchens’. With no physical store or dine in options and no physical contact with consumers, dark kitchens provide a cheaper alternative to the traditional brick and mortar restaurants (Khan, 2020; Kulshreshtha and Sharma, 2022). Dark kitchens are also able to operate at reduced operational costs since they do not need additional staff such as front service staff or cashiers. This cost saving measure allows dark kitchens to pass on the benefits to customers through reduced pricing (Hakim et al., 2023; Kulshreshtha and Sharma, 2022).

While the concept of dark kitchens has existed for many years (Hakim et al., 2023), this growth has been propelled by the COVID-19 pandemic (Kulshreshtha and Sharma, 2022; Rinaldi et al., 2022; Shapiro, 2023; da Cunha et al., 2024). At a time when safety concerns prompted lockdowns and social distancing measures there were temporary closures of hospitality venues and consumers became heavily reliant upon third party food delivery services like UberEats, Just Eat and Deliveroo (Giousmpasoglou et al., 2023). These platforms, which are exclusively available online through their websites and mobile applications, partner with local food businesses to collect and deliver meals to customers using delivery riders or drivers. In the UK dark kitchens have proven quite successful. Since March 2020, there has been a 70% increase in the average order volume per dark kitchen according to Deliveroo – one of the leading dark kitchen operators in the UK (Magnet, 2021). It has been estimated that more than 750 dark kitchens are operating in the UK in 2020, but the actual number of this food model service remains unknown, especially after the pandemic (Savills, 2022). Consumers are clearly appreciating the convenience of ordering meals from dark kitchens and as such, there is a continued demand for delivery and takeout services causing this trend to persist post-pandemic (Rinaldi et al., 2022). This significant change in consumer behaviour and increasing demand for delivery and takeaway options over in person dining, has consequently raised the profile of dark kitchens within the food industry.

The number of published studies on dark kitchens has increased in recent years, driven by the growing popularity of the dark kitchen operational model. Most studies were focused on consumer’s perceptions of dark kitchens (Cai et al., 2022; Hakim et al., 2022), characteristics and typologies (Ashton et al., 2022; da Cunha et al., 2024; Hakim et al. 2023; Rinaldi et al. al. 2022), factors contributing to their development and success (Vu et al., 2023) as well as their economic, social and environmental impacts (Li et al. 2020). In Hakim et al. (2022), the study identified the lack of public awareness of what dark kitchens were and factors influencing their purchase intention from such food businesses. Meanwhile, Cai et al. (2022) revealed that consumers prefer the convenience and variety of food options provided through this business model but were also concern with the employee welfare and working conditions in dark kitchens. The concerns regarding the working conditions of dark kitchens were that they do not meet the minimum industry standards in terms of kitchen operations (Cai et al., 2022; Giousmpasoglou et al., 2023). The characteristics of dark kitchens revealed different models of dark kitchens such as independent dark kitchens and shared dark kitchens (Hakim et al., 2023) with fast foods (Rinaldi et al., 2022), utilitarian meals, snacks and desserts (Hakim et al., 2023) being the most common type of foods sold by dark kitchens. Li et al. (2020) highlighted the public health impacts associated with dark kitchens such as the increased availability and accessibility of unhealthy food options around the clock.

### 1.1 Research Gap

While the significant phenomenon that is dark kitchens has provided a new way for consumers to dine and interact with restaurants in a post-pandemic world, this concept does present challenges for both consumers and environmental health officers (EHOs). Consumers often find it difficult to identify dark kitchens from a standard restaurant in food delivery apps (Hakim et al., 2023). Online delivery platforms allow food businesses to sell their food under multiple brand names if they are offering different menus. This can potentially be misleading for the consumers, who think that these are all menu options from different restaurants. As such, on many occasions, customers ordering food from dark kitchens are unaware of where and how the food is produced and, in many cases, (for instance dark kitchens operating exclusively through social media) are left with no way of contacting the restaurant/owner (Eccles, 2021; Giousmpasoglou et al., 2023). Furthermore, there are an increasing number of new businesses operating as dark kitchens. These are often difficult to identify due to their lack of visibility and therefore makes it easier for them to operate under the radar without the oversight of the competent authority. This lack of transparency and the food business’ failure to comply poses challenges for EHOs, who have both limited resources and time. These issues highlight the first critical research gap that our study will address, i.e., to understand the challenges associated with identifying dark kitchens and exploring potential solutions to improve its transparency. This led to our first research question: ‘How do Local Authorities identify dark kitchens?’

The control of food safety including allergen and cross-contamination management in dark kitchens can be difficult, particularly where shared spaces are being used. Previous studies had reported preliminary findings on consumers’ concern with the cleanliness and hygiene standards of dark kitchens (Cai et al., 2022) and that perceived food safety was identified as one of the factors affecting consumers’ willingness to purchase from dark kitchens (Hakim et al., 2022). However, there remains a lack of studies in relation to the challenges associated with inspecting and managing food safety in dark kitchens from the perspectives of EHOs and dark kitchens. This led to our second research question: ‘What are the challenges and opportunities for food safety inspections and implementation in dark kitchens?’ Given that dark kitchens are expected to shape the future of food delivery services, further research is warranted to address both the opportunities and challenges associated with this type of food service model. This study aims to assess the challenges in identifying and regulating dark kitchens and to identify potential interventions to increase food safety compliance in dark kitchens by working with LAs and dark kitchen owners and tenants.

## 2 Methods

This study utilised a mixed method approach including online survey, focus group discussions and semi-structured interviews to examine the challenges and opportunities for local authorities and dark kitchens in ensuring food safety. Principal Component Analysis and thematic analysis were used to analyse the quantitative and qualitative findings. According to Carugi (2016), the findings of a study are more valid when different methods of data collection and analysis converge on the same conclusion. It also provides a broader and a multi-dimensional perspective of a phenomenon as the weakness of a single method is mitigated, thus increasing the reliability and validity in the findings (Kopinak, 1999). Specifically, within our study, combining survey, focus group discussions with EHOs and semi-structured interviews with dark kitchen operators provided a more comprehensive understanding of the overall findings. The initial quantitative findings were further explored through focus groups and semi-structured interviews to understand why such challenges exist and how these could be addressed. The focus group discussions also facilitated EHOs’ interaction and revealed shared experiences faced by EHOs from different local authorities.

### 2.1 Questionnaire and Topic Guides Development

The questionnaire and topic guides were designed to answer two main research questions i.e., (i) How do Local Authorities identify dark kitchens? (ii) What are the challenges and opportunities for food safety inspections and implementation in dark kitchens? Prior to developing the questionnaire and topic guides, the study team posed the two main research questions in the online forum ‘Knowledge Hub’ for local authorities and trading standards officers in England about food safety in dark kitchens. We received responses from the forum including difficulties in identifying dark kitchens that were not registered, challenges of inspection and awarding food hygiene rating due to multiple dark kitchens sharing the same venue and challenges of inspecting dark kitchens with multiple trading names at the same address. Based on the issues discussed above, the study team developed the questions and then carried out a pilot-test of the questionnaire. The following dark kitchen definition was used in the questionnaire: ‘Dark kitchens are food services without front-facing service or direct contact with customers and offer meals purchased by online delivery. Home-based, rented or shared premises will be included in this context’. Based on the feedback provided by the EHOs in the pilot-test, additional options i.e., ‘Difficulty identifying location of unregistered dark kitchens’ and ‘Dark kitchen operators unaware of their obligation to register’ were added to the following question ‘What are the challenges faced by your LA to identify dark kitchens? Please select all that apply.’ An additional option, i.e., ‘allocating a food hygiene rating to the dark kitchens’ was added to Question 11: What are the challenges faced by your LA when inspecting a dark kitchen?’.

Similarly, the focus group discussion topic guide with EHOs and semi-structured interview topic guides with dark kitchen operators were checked for face and content validity with the research team. For face validity, the research team reviewed the guides for clarity and whether the questions were suitable for participants. For content validity, the topic guides were evaluated by the research team and several EHO contacts and one dark kitchen operator to ensure the guides captured all relevant aspects of the challenges and opportunities in ensuring food safety in dark kitchens. Additionally, the topic guides were also shared with our Patient and Public Involvement and Engagement (PPIE) group with representatives from the LA and a food allergy patient. The questionnaire and topic guides are available in Supplementary Materials 1 – 3. The study received ethic NHS Health Research Authority approval from London – Fulham Research Ethics Committee (24/PR/0280). All participants were provided with a £30 Amazon gift voucher.

### 2.2 Online Survey

The questionnaire was uploaded onto onlinesurveys.jisc.ac.uk. The cross-sectional online survey was conducted among EHOs based in LAs in England. The online survey was distributed through the Local Government Association (LGA) and Environmental Health Officers’ online forum. A sample size of 156 was required based on 95% significance level, 5% margin of error, population size of 317 [there are 317 Local Authorities in England (GOV.UK, 2023)] and 10% non-response rate.

### 2.3 Focus Group Discussion

Two focus group discussions with 8 participants per group were conducted to generate a richer qualitative dataset to help understand EHOs’ experience and insights of food safety in dark kitchens. Examples of questions include ‘Could you share your experiences or challenges when inspecting a dark kitchen?’, ‘How do you identify dark kitchens?’ and ‘How could we improve the food hygiene inspections of dark kitchens?’ EHOs were recruited through the online survey. Each focus group discussion was conducted online using MS Teams and lasted 60 – 75 minutes. To begin the focus group, an overall outline of the research project and the aim of the research was mentioned. The confidential nature of the project and the participation being voluntary was also emphasised at this point. Each focus group session was recorded and transcribed using MS Teams and immediately after the sessions, transcriptions were checked against the audio recording to ensure accuracy.

### 2.4 Online Semi-Structured Interviews

To obtain a richer understanding of the experiences of dark kitchen operators, we also conducted a series of semi-structured interviews. Examples of questions include ‘How do you ensure the food safety of your dark kitchens?’ and ‘Have there been any challenges for food safety inspections of dark kitchens?’ A total of 16 semi-structured interviews were conducted. This included 12 dark kitchen tenants and 4 dark kitchen owners, of which 2 were home-based. The list of dark kitchen participants’ demographic characteristics is provided in Supplementary Material 4. Our study defines dark kitchen tenants as food business operators that leased kitchen space from a land agent, property agent or other restaurants rather than owning the premises themselves. Dark kitchen owners and tenants were all recruited through the Facebook social media platform. Each interview lasted between 30 to 45 minutes and were conducted online via MS Teams. Participants were again provided with an overall outline of the research project and the aims. The confidentiality of the research and the voluntary participation were again emphasised to each participant prior to starting the interviews. The online interviews were recorded and transcribed using MS Teams and upon completion of the interviews, were immediately checked for accuracy against the audio recording.

### 2.5 Data analysis

Survey data were analysed using descriptive tests. A Principal Component Analysis (PCA) was done to to reduce the dimensionality of a data set while retaining the most important information. Indicators with factor loadings higher than 0.50 were retained (Cheung et al., 2023). Components with eigenvalues greater than 1 were retained as they contribute substantially to the overall variability. The sample adequacy was measured using Kaiser-Meyer-Olkin > 0.60. SPSS Version 29.0 was used with all categorical variables expressed as numbers and percentages. For the qualitative questions in the survey, thematic analysis was employed. The responses were reviewed systematically, and the data was coded and then organised into the most relevant themes.

#### 2.5.1 Thematic analysis of focus group discussions and interview transcripts

The audio recordings of the focus group discussions and interviews were transcribed. For both the focus groups and interviews, thematic analysis was used to analyse the data. All thematic analysis was based on Braun and Clarkes six step framework (Braun and Clarke, 2006; 2022). In the initial step, transcripts were read and re-read to familiarise with the data. The second step involved producing initial codes from the data. This was achieved using NVivo version 14. All transcribed data was input into NVivo and organised into meaningful groups i.e. coded. Each transcribed document was systematically worked through, without overlooking any of the data. An inductive coding (bottom up) approach was used. The purpose of this phase was to reduce the data into a more manageable format. This led to the third step, i.e., searching for themes, where the initial codes were placed into potential themes that captured something interesting or significant in relation to the research questions. At this stage, a preliminary thematic map was created highlighting the emergence of initial themes. The fourth step looked at reviewing these initial themes and refining them to produce overarching themes and subthemes. This involved the re-reading of all transcribed data to ensure nothing was missed. In step 5, the existing themes were clearly defined and further refined to capture the essence of the theme. Following the analysis, a final thematic map was created for each of the questions to provide a visual representation of the themes. The final stage was the write-up of the findings.

## 3 Results

The findings of this study are broadly structured as follows. Firstly, the study explores dark kitchen operators’ perspectives on the challenges in maintaining food safety and their proposed strategies to improve food safety compliance. Secondly, our study examines EHOs’ perspectives on the challenges faced in identifying and inspecting dark kitchens and highlights the potential opportunities to address these challenges.

### 3.1 Dark kitchens’ Perspectives

Four dark kitchen owners (including 2 home-based) and 12 dark kitchen tenants took part in the semi-structured interviews between April and May 2024 to share their insights. Sixteen semi-structured interviews enabled us to achieve data saturation where no new themes were identified. This aligns with Guest et al. (2006) where up to 12 interviews would enable data saturation. Section 3.1.1 highlights the challenges faced by dark kitchens followed by Section 3.1.2 which details the strategies to address them.

#### 3.1.1 Challenges Faced by Dark Kitchens

##### 3.1.1a Managing food safety in shared spaces

Many of the dark kitchen operators noted the difficulty in managing food safety in shared dark kitchens. There were struggles in maintaining hygiene due to shared resources, conflicts over responsibility pertaining to pest control and issues with staff compliance and cooperation. Collectively these issues can impact the ability to effectively maintain food safety standards and, in some instances, participants mentioned how these difficulties prompted them to relocate or establish their own independent dark kitchen. The challenges highlighted in maintaining food safety in dark kitchens stresses the need for more robust management systems to allow for operational harmony in shared kitchen environments.

*You know, because sharing resources, so we had competition for kitchen equipment, storage space and all this. And sometime this results in temporary conflicts in coordinating operations and also there is limited controls. It gave me limited control over the kitchen environment, including cleaning standards. (DK 13, Owner, London)*

*That was one of the reasons I had to relocate. The person I was sharing with was not that hygienic and wasn’t really cooperating at times… we had a lot of issues, both pests, rodents a whole lot of things. (DK 11, Tenant, Portsmouth)*

##### 3.1.1b Food safety handling during delivery

Dark kitchens are heavily reliant upon delivery drivers/riders for transporting their food items. Dark kitchen owners and tenants repeatedly mentioned how the food handling practices of delivery drivers/riders were concerning. Participants highlighted how on many occasions; food was often delivered in compromised conditions as mentioned through complaints by customers. There is a lack of transparency in the food handling practices of delivery drivers/riders who potentially have inadequate food safety standards and hygiene practices.

*I’ve had issues where we get complaints from customers saying that the food got to them in different states than it was packaged. I don’t know what’s happening between when the food left me to when it got to them. (DK 6, Tenant, Southampton)*

*I make sure that the food is hygienic by washing my hands, wearing gloves and I give it to someone that has been on the road for probably more than four hours, hasn’t even washed his hands and he’s getting this food and exposing it in the dispatch bag where I’m not sure whether it was washed this morning or it wasn’t washed this morning. And then when he was stuck in traffic, making the other stops. (DK 6, Tenant, Southampton)*

##### 3.1.1c Staff

The final challenge mentioned by dark kitchen operators when operating dark kitchens was issues relating to staff, especially finding trained personnel who had relevant experience. Similar to other food services, the dark kitchen operational model faces a high turnover of staff, which can frequently disrupt operations and will necessitate constant training, which was further expressed by participants.

*Somebody might order something, and they end up doing the wrong thing. So that ends up being a waste. You have to do it all over again and it’s wasting resources… (DK 2, Tenant, London)*

*The staffs are not properly trained. They are not experienced. They are also leaving constantly. So that’s also a problem… Often times I have to train the staff myself. (DK 9, Tenant, Bristol)*

##### 3.1.1d Delays in updating online platforms

One of the main issues was the dependence on online aggregators for updating and communicating with customers through their platforms. Participants highlighted an increased lag time in providing updates concerning allergen information or menu changes, ranging from as little as a few hours to several days. A delay in updating allergen information on online platform may result in consumers, especially food hypersensitive consumers not having updated access to information such as the change in recipe, especially if food allergens were used. As a consequence, many dark kitchen owners and tenants preferred using their social media channels to quickly update and inform customers of any changes.

*It takes longer time for it to be updated – around 2 days. That’s why I don’t completely rely on them. I also make my updates on social media handles that way. I’m directly informing my customers on the new developments. (DK 9, Tenant, Bristol)*

*Usually allergen free food issues where our delivery platforms are not really swift in updating and also making sure that information is being circulated so that is also a major issue. (DK 16, Home-Based, Peterborough)*

#### 3.1.2 Strategies to Improve Food Safety Compliance in Dark Kitchens

Two key strategies were shared by dark kitchen operators to address some of the challenges identified above. This includes training and more frequent food hygiene inspections.

##### 3.1.2a Training

Both dark kitchen owners and tenants acknowledged the need for more formal training to enhance their education and awareness, consequently improving food safety standards. Specifically, dark kitchen operators mentioned the need for mandatory training for not only themselves, but also for delivery drivers and riders. They expressed concerns about the handling of food once it had left their premises and felt it essential to provide food safety training and hygiene education to delivery drivers/riders.

*I think that there should be free online trainings, because I have to pay for my staff sometimes to be able to get access to this particular training and because of the cost, it might limit some people from actually being able to get to know new things or learn (DK 8, Owner and Tenant, London, Manchester and Birmingham)*

*The delivery services, sometimes they do not practice hygiene. We try to practise as much hygiene as possible, but this delivery service people we hand this food over to, do they know what hygiene is? Do they practise any kind of hygiene? Do they even know what food safety is? (DK 3, Tenant, London)*

##### 3.1.2b Inspections

Dark kitchen owners and tenants expressed a need for more frequent inspections to enhance their food safety standards. They appreciated the importance of regular inspections and believed them to serve as a catalyst to enhance their practices and adherence to food safety protocols and elevate their individual standards.

*I think inspections should become more frequent than it is. So, I get inspected once a year and I feel that that’s way too little because I deal with people every day and I’m responsible for peoples lives and something could go terribly wrong within that period that no one is checking to make sure everything is OK. (DK 14, Home-Based, Liverpool)*

*Increase the number of times for visits because if I know that, OK in a month, I will be paid a visit twice. I would really be up doing and be very careful and take more precaution. I think that would also be helpful (DK 16, Home-Based, Peterborough)*

### 3.2 Local Authorities’ Perspectives (Quantitative findings)

**Figure 1:**
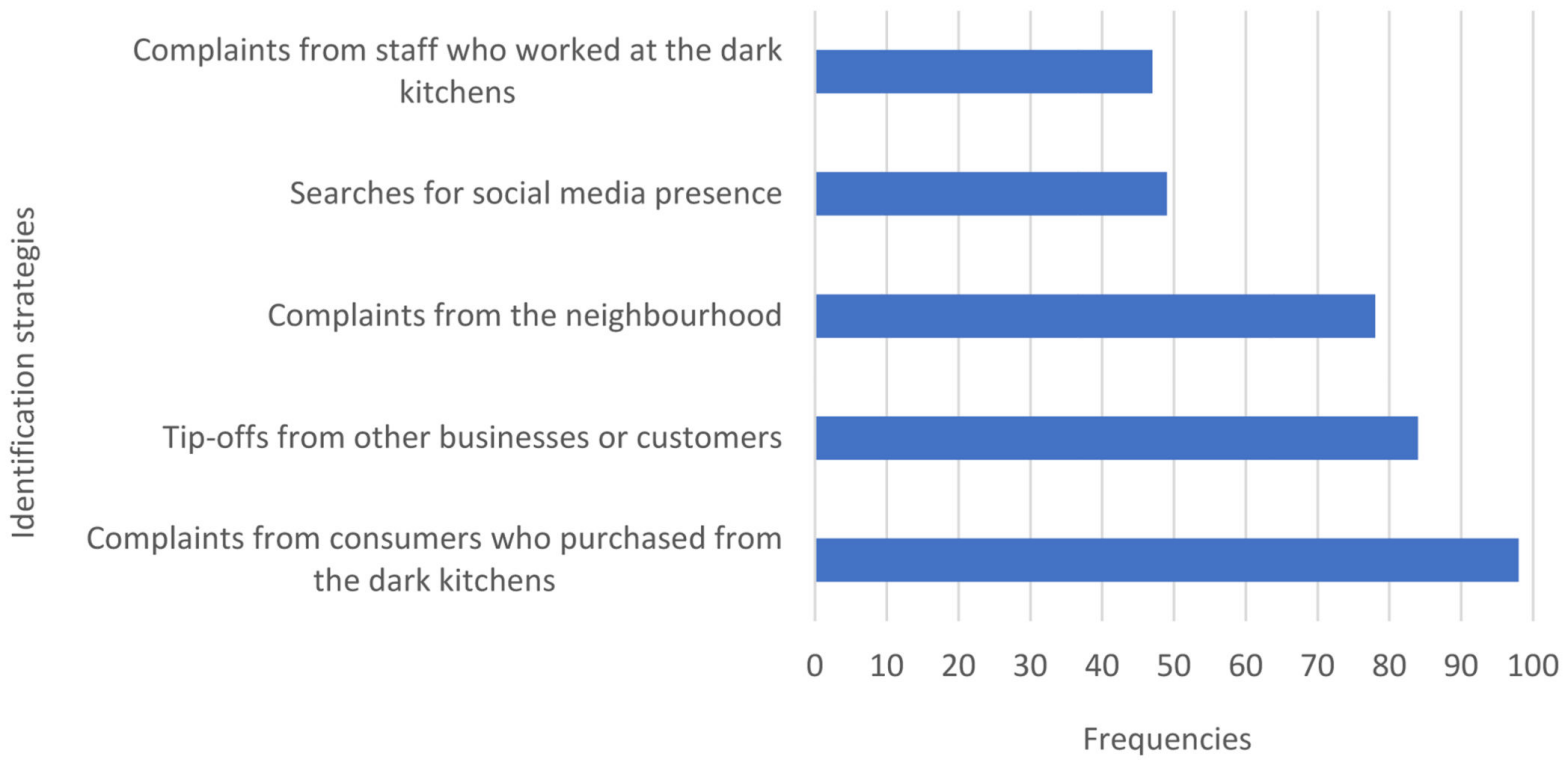
How do local authorities identify unregistered dark kitchens? (n=123)

A total of 123 valid responses were received from 91 local authorities in England. Approximately 60% of the participants had more than 15 years of experience working as an EHO. The majority have inspected a dark kitchen and faced challenges when it comes to identifying the dark kitchens (>75%). More than 60% of the respondents were not able to proactively search for unregistered dark kitchens in their local authorities (Table 1) but instead rely on customer complaints, tip-offs from other businesses or complaints from the neighbourhoods before they become aware of their existence (Figure 1).

Three components explained the challenges faced by local authorities in identifying dark kitchens (58.68% of variance). The first component was about dark kitchen invisibility. The second factor was inadequate human and financial resources of LAs, while the third factor concerns the challenges associated with the registration of dark kitchens and difficulties about understanding what dark kitchens really is. Some of the key challenges faced by EHOs in identifying dark kitchens were due to dark kitchens operating under several trading or brand names (77.2%), lack of staff to proactively look for them (65.8%) and dark kitchen operators unaware of their obligation to register (64.2%) (Table 2). Besides facing the challenge of identifying and locating dark kitchens, the local authorities were also faced with challenges when inspecting the dark kitchens. Two components explained the challenges faced by the local authorities when inspecting a dark kitchen (62.4% of the variance) (Table 3). The first component refers to the shared-space nature of the dark kitchens. The biggest challenge identified under this component was the uncertain or sporadic operating hours which make it difficult for EHOs to visit and inspect the DKs. Another main challenge was the inspection of shared dark kitchen space. For example, several different food business operators that use the same DK space at the same time. This makes the inspection more challenging, especially in determining responsibilities and how staff from different food business operations (FBOs) ensure hygiene, especially in communal spaces. The second component is less clearly defined, but includes factors such as dark kitchens acting as middlemen by buying and re-selling food from other food businesses, and the awarding of food hygiene ratings to these establishments.

### 3.3 Local Authorities’ Perspectives (Qualitative Findings)

#### 3.3.1 Challenges in Identifying and Inspecting Dark Kitchens

Sixteen EHOs took part in two online focus group discussions in April – May 2024. These discussions provided a deeper understanding of the challenges associated with identifying and inspecting dark kitchens as well as opportunities for improvement in these processes.

##### 3.3.1a Resource constraints

EHOs highlighted how resource constraints impacted their ability to identify and inspect dark kitchens. A common issue with dark kitchens is multiple registrations for the same food business, which leads to administrative burdens consuming valuable time and resources. EHOs also highlighted how they are often understaffed to proactively search for dark kitchens and consequently inspect them. EHOs additionally mentioned the challenge in tracking down the many businesses that use social media to interact with their customer base due to limited resources and the lack of official accounts to interact with these food businesses.

*Lack of resources to proactively look for them most difficult challenge. (EHO, survey response)*

*So, there’s a bit of investigation work to see if they’re registered… you know, it depends if you’ve got the time and the resources to be able to do all that it does, you know, it’s not always the first thing that we’re working on. (EHO 4, Female)*

*Unless we’re using our own private accounts, we can’t go looking for them… (EHO 5, Female)*.

Additionally, where multiple dark kitchens operate from one location, it necessitates multiple staff members to be available which is difficult, particularly for the many dark kitchens that operate in the evenings.

*Ideally, we want to inspect all at the same time to save on trips to the venue as it can be 4+ businesses in one space who register at the same time, but this normally requires having 2+ staff members available on the same day or it might need an evening inspection if it opens late, which is even harder to co-ordinate. (EHO, survey response)*

Additionally, EHOs mentioned how challenges regarding allergens are compounded by resource constraints. In some local authorities, allergen regulation is enforced separately by Trading Standards Officers (TSOs). Like EHOs, trading standard officers faces similar resource challenges.

*In terms of officers and you know, trading standards officers… they’re brilliant, but I think they’re overstretched (EHO 2, Female)*

##### 3.3.1b Lack of visibility

The lack of visibility of dark kitchens further poses a challenge for EHOs as often food businesses may appear closed when in actual fact, they have transitioned to non-customer facing operations. Since many dark kitchens operate without a front-facing store, they will not appear accessible to the public.

*It’s the unknown that’s the problem, isn’t it? It’s the unknown scale of it. We don’t even know what is out there. (EHO 12, Male)*

*Businesses can now looked “shut” as they decided to no longer have a customer facing operation, so we close them on the system as we thought business has closed. (EHO, survey response)*

EHOs also mentioned how dark kitchens sometimes obscured their true location, by initially registering at commercial premises but subsequently utilise unregistered residential kitchens and operate from unknown locations.

*They’ve had a visit from an online aggregator to give them the once over to say yes, this FBO is OK for running the business from, but then the FBO never opens the doors commercially, give back the lease or whatever it is, and then go and cook it in a kitchen from somebody’s house or their own home. (EHO 1, Female)*

This lack of visibility further extends to the increased use of social media platforms. Dark kitchens may bypass formal registration and inspection by solely advertising their food business through social media, where they are not required to disclose their physical location. Sometimes, incorrect or even no information is provided by dark kitchens during the registration process. This lack of clarity made it difficult for EHOs to identify and effectively monitor and enforce food safety standards.

*They pop up with no information… and incorrect information put in at registration. (EHO, survey response)*

Despite efforts from EHOs to contact these food businesses, EHOs were sometimes met with resistance, such as vague responses, blocked accounts and refusal to disclose their operational details. Some EHOs also reported that dark kitchens may avoid EHO identification by pretending to use existing but closed business sites. Consequently, this adds an extra layer of difficulty for EHOs to track and regulate these dark kitchens.

*We found them again on social media and then when I’ve chased these up*… *they would not say where they were based. (EHO 2, Female)*

*Dark kitchens that turn out to be people working from home are also problematic. They advertise on social media, however when you contact them to alert them of their obligations, they block you. (EHO, survey response)*

##### 3.3.1c Multiple trading names

Dark kitchens may use multiple trading names for their food business, which further presents significant challenge in identifying their location. For instance, the use of multiple trading names led to confusion among EHOs, who encountered duplicate registrations for the same food businesses.

*The big chains like XYZ who are registering more than one business operating out of one address even though it’s the same FBO. And so consequently we’ve got duplicate registrations. (EHO 1, Female)*

EHOs also highlighted limitations in their current databases when recording multiple trading names. This can lead to incomplete or inaccurate records, which can reduce the ability of EHOs to identify and inspect dark kitchen operations.

*We have an issue where we can have more than two names, but there’s a character limit… We’ve had people wanting 10/12 names which we just can’t handle.’ (EHO 14, Male)*

EHOs further mentioned how the presence of multiple trading names can create difficulty during inspections in establishing the true ownership and operation of a food business, which can then lead to issues in ensuring their compliance with regulations.

*There are many trade platforms that businesses are now trading under. Which gives you that level of uncertainty about exactly who is controlling this as an activity or be it from one single kitchen. (EHO 11, Male)*

##### 3.3.1d Lack of guidance

Another challenge experienced by EHOs when inspecting dark kitchens, was the absence of clear directions from the regulatory body. EHOs felt that insufficient guidance hindered their ability to effectively enforce food safety standards and led to inconsistent enforcement practices across local authorities. This then creates ambiguity among EHOs as to what is deemed as safe food handling practices, especially when it comes to home-based operations.

*And again, I do think the regulatory body has a role to play in you know in all of this and need again need to get more direction from them and more involvement needs to be discussed. (EHO, Female)*

*And then the other thing I was just wanted to mention was inconsistencies amongst local authorities as to what is deemed safe to do at home… I think sometimes there’s an idea amongst the industry that if you’re doing it from home, it’ll almost sort of bypasses legislation and it obviously it doesn’t. (EHO 14, Male)*

##### 3.3.1e Communication difficulties

EHOs also experienced communication difficulties that manifest in establishing ownership of the dark kitchens themselves, which is further exacerbated by staff ambiguity regarding responsibility for communal spaces. Language barriers presented additional complications. Staff working in dark kitchens sometimes lack proficient English, which in turn impedes effective communication and the inspection process. Language barriers also pose a challenge for inspecting premises if the officer cannot read what is being sold.

*There are problems identifying the food business operator, difficult to identify the roles and responsibility and its time consuming… sometimes there is a denial about responsibility for communal spaces that may directly affect the business operation’ (EHO, survey response)*

*Language barrier - A lot of the Chinese dark kitchens only operate on the ‘Hungry Panda’ app which is only for Chinese speaking people. Establishing ownership details is challenging, and engaging with staff who can’t speak English. (EHO, survey response)*

##### 3.3.1f Hygiene and food safety standards

When inspecting dark kitchens, EHOs reported having experienced various problems relating to hygiene and food safety standards. These challenges included challenging working conditions such as cramped spaces and structural issues like lack of ventilation and hot water.

*Major structural issues (as cooking in a cupboard) with no ventilation, no hot water to wash hand basin and multiple extension leads that were full of other cooking and cold holding equipment plugs. So, no socket space for an electric hot water heater… (EHO, survey response)*

EHOs also emphasised how the inadequate cleaning practices of dark kitchens in relations to allergens will potentially cause cross-contamination - in particular where multiple food businesses are operating from the same premises. Staff may often be unaware of cleaning protocols in other businesses and this lack of awareness may lead to compromising food safety standards.

*If you’ve got two businesses operating from one kitchen and neither knows necessarily what the other one’s doing, what food they’re handling, how they’re cleaning or whatever, I think cross contamination could be an issue. (EHO 6, Female)*

Furthermore, EHOs reported the challenges administering the Food Hygiene Rating Scheme in dark kitchen operations, making it difficult for consumers to assess the hygiene rating of the food business.

*Food Hygiene rating issues… I feel that in many cases the original registered business has received a low rating and thus not allowed to trade on the platforms - they often don’t want to pay for a re-score and so rebrand with a different name at the same premise with the same FBO but become unrated and thus get a ‘free’ rating. (EHO, survey response)*

#### 3.3.2 Strategies to Identify and Inspect Dark Kitchens

##### 3.3.2a Staff and Resources

The lack of resources is a recurring challenge to proactively search for and identify dark kitchens. Many EHOs highlighted the need for more funding and staff to conduct thorough online checks and the ability to use social media for investigations.

*LA need more staff/resources…More funding for officers to carry out proactive work such as searching through online food platforms to gather information about who is operating in the area. (EHO, survey response)*

Currently, when identifying dark kitchens, EHOs primarily use three main methods. Firstly, routine inspections of premises and using planning applications helped to reveal the existence of dark kitchens. Secondly, complaints from staff who worked in dark kitchens or customers of these establishments often serve as valuable leads for EHOs, alerting them to the presence of unregistered dark kitchens. Finally, social media platforms play a vital role in identifying dark kitchens. This included searching popular online delivery aggregators including Just Eat, UberEats and Deliveroo and checking that businesses are listed on the Foods Standards Agency (FSA) website before being allowed to trade in these platforms. Where possible, EHOs will monitor these platforms for advertisements or even complaints related to unregistered food businesses. Some LAs also created official accounts to communicate with food businesses.

*Sometimes staff, they’ll complain about the conditions and maybe they’ve been sacked and they just like you know, I’m going to dob in my boss sort of thing… and customers, maybe they’ve had some food they didn’t like or suspected food poisoning. That’s normally how we find out about new ones. (EHO 13, Female)*

*Every time we do a trawl of Just Eat, we will find businesses or business names that we didn’t know about and that really frustrates me. (EHO 15, Female)*

Similarly, increasing resources through both funding and staffing was repeatedly highlighted by EHOs as an essential means of improving the food hygiene inspections of dark kitchens. More EHOs and increased funding will allow for more frequent and thorough inspections and would enable more proactive searching for unregistered dark kitchens.

*More funding for LAs/EH teams to increase staffing levels. This would enable more time for proactive searching and attempts to access a business unannounced (EHO, survey response)*

##### 3.3.2b Guidance and enforcement

The EHOs emphasised the need for guidance and stronger regulations from the regulatory body to improve the food hygiene inspections of dark kitchens. EHOs mentioned revising registration forms to ensure dark kitchens include details of whether they operate under other trading names. This in turn help to ensure all aspects of their operations are disclosed and allow for better monitoring and regulation. This also ties in with the suggestion for mandatory licensing schemes and increased penalties for non-compliance to deter businesses from operating without registration.

*By asking more details of the roles, responsibility, operations of the dark kitchen during registration. (EHO, survey response)*

EHOs emphasised the need for comprehensive and specific guidelines tailored to the unique operational structure of dark kitchens. EHOs mentioned clearer instructions on evaluating hygiene practices, allergen control, and the structural standards in shared facilities are needed. This tailored guidance will assist EHOs in conducting thorough inspections, mitigating inconsistencies and ensuring a standardised approach is met across all local authorities. Guidance should also be available for dark kitchen operators and providing them with support and knowledge of having to meet certain legal requirements, to ensure they do not operate without registration and are adhering to food safety standards.

*More guidance from FSA on different scenario’s… Need standardised approach by all local authorities. (EHO, survey response)*

*Be good to have guidance around shared units and managing hygiene and allergens safely and general standards/requirements/best practice. (EHO, survey response)*

*Having to meet certain legal requirements before being able to register a food business would ensure that FBOs cannot register without providing specific information and details about their food business. (EHO, survey response)*

To address the issue of unregistered dark kitchens, EHOs suggested the need for increased regulation and enforcement. This primarily included the requirement of all food businesses to obtain a license prior to operating.

*Licence the business… formal licencing long overdue. Should have been coming long, long time ago and that’s the only real effective way for local authorities to manage this effectively. (EHO 11, Male)*

EHOs further suggested implementing fixed penalty notices as a deterrent for businesses that fail to register. This approach would create a financial consequence for non-compliance, which would encourage businesses to adhere to registration requirements.

*FPNs, fixed penalty notices for not registering… Ultimately, there needs to be some sort of penalty if people aren’t (complying). (EHO 10, Female)*

*Fixed Penalty Notice to be paid to cover administration costs for operating without a licence and the business being unable to open until they are licenced. (EHO, survey response)*

##### 3.3.2c Collaboration and engagement with various stakeholders

EHOs suggest engagement and open communication with food businesses themselves to offer valuable guidance and support to ensure their compliance with regulations. This may increase rapport between EHOs and dark kitchen operators that ultimately promote safer food practices and allow them to thrive.

*We say right, we’ve found you, we’ve tracked you down. However look, let’s work together. Let’s have an open policy as we want you to do the right thing. We recognise that you want to do the right thing. Let’s try and get things right. (EHO 11, Male)*

EHOs work closely with trading standard officers when it comes to ensuring effective allergen control. While EHOs will often deal with issues of cross contamination themselves, issues relating to allergens are often dealt with by trading standard officers through collaboration. This collaboration includes both referrals from EHOs to trading standard officers for further investigation, as well as joint visits whereby each department contributes their expertise. This partnership between EHOs and trading standard officers is key in ensuring the maintenance of effective allergen control in dark kitchens.

*We will report any allergen concerns to Trading Standards although we would deal with cross contamination… (EHO 4, Female)*.

*They do joint visits with us, a lot of advice and guidance… (EHO 7, Male)*

Building owners and landagents were identified as other potential stakeholders who could support Local Authorities. They play a crucial role in leasing out commercial properties which include dark kitchen spaces. Collaborating with landagents can therefore provide EHOs with insight into any activities or unregistered operations by tenants.

*We engage with building owners to establish the businesses trading in each unit to check on registration… (EHO survey response)*

Finally, EHOs mentioned working closely with online delivery platforms. Online aggregators have extensive information on businesses operating on their platforms. Through engagement with these online aggregators, EHOs can gain access to information about food businesses listed on their platforms. The online aggregators can also share information about any new businesses joining their platform, which can allow local authorities to verify their registration status. This collaboration can ensure that EHOs are up to date and informed of any newly registered dark kitchens that are operating through these platforms, allowing EHOs to inspect and regulate them.

*I would recommend that the food platform they are selling from gives an address for all business or if they informed the local authority of any new food businesses operating on the platform. (EHO, survey response)*

*I think it would help if they shared information with a local authority before they onboarded the business. (EHO 7, Male)*

EHOs additionally suggested a need for online aggregators to play a more active role in verifying that businesses who are registered with their platforms comply with food safety regulation and are registered with the authorities. This would significantly assist in identifying inadequate food safety practices and unregistered operations.

*There needs to be some sort of control… You know the the businesses on there, they say that they are checking and only allowing registered businesses on. But the reality is that they’re somehow getting on. So, I think we need to, it feels like it’s a due diligence. (EHO 6, Female)*

## 4 Discussion

Dark kitchens are an attractive method of operating a food business and made possible by the increase in use of online food delivery applications. As they are still fundamentally a catering business, they share many of the problems of other takeaways and restaurants such as lack of staff and access to appropriate training. Similarly, enforcement officers often encounter many of the same problems they find in other food business operations. However, dark kitchen also present some additional challenges to both the operators and the enforcement bodies.

One major hindrance of the dark kitchen operational model was identification of the dark kitchens. While no previous literature has outlined the challenges of identifying dark kitchens, EHOs in both the survey and focus groups voiced similar thoughts. This suggests that the challenges associated with identifying dark kitchens are a fundamental issue of the operational model itself. The difficulty in identification may pose substantial barriers in the effective monitoring and regulation of food safety procedures. This underscores the need for further investigation into the challenges of identifying dark kitchens. Traditionally food businesses were located in areas that attracted passing trade and were visible. Online delivery applications mean that this is no longer strictly necessary, and businesses can relocate to less obvious premises (Hakim et al., 2023). There is currently a legal requirement for all food businesses to register with the local authority in which they are based, but if a food business does not comply with this requirement the EHO has to identify them. The current methods used by EHOs when identifying unregistered dark kitchens were outlined in this study. While these assisted EHOs in identifying dark kitchens, they are also limited. For example, it may not always be possible to identify the exact location due to dark kitchens often operating in non-traditional spaces and having irregular operating hours (Hakim et al., 2023; da Cunha et al., 2024). Similarly, while complaints from staff and customers can provide essential leads for EHOs in identifying unregistered dark kitchens, these are dependent on their accuracy and timeliness with delays and incomplete information likely to hinder EHOs ability to act promptly. Dark kitchens also frequently use social media (Ghazanfar et al., 2023) and often EHOs do not have the resources or channels to monitor such platforms.

In May 2021 the FSA released a report acknowledging that the rise of dark kitchens presents significant complexities for food safety enforcement and warrant further research to comprehensively address these challenges (Foods Standards Agency, 2021). This acknowledgement by the FSA aligns with the findings from this research where EHOs mention a dissatisfaction in the current guidelines being provided. While the report released by the FSA showcases their commitment to provide change, it is clear from the results of this study that there is room for improvement. Both licencing and fixed penalty notices were repeatedly emphasised by EHOs with these measures providing a financial motivation to adhere to regulations. Additionally, sanctions can ensure that FBOs that have saved costs through non-compliance do not gain unfair advantage over businesses that are fully compliant (Macrory, 2006). These findings align with previous research which emphasise the importance of enforcement in maintaining food safety standards (Meyer et al., 2017). In 2018, the FSA released a report reviewing the food law code to date in efforts to implement change. The document supports the notion of incentives such as fixed penalty notices (FPNs) for failure to comply with registration and standards. However, it further indicates that additional research is needed into these areas before implementation (Foods Standards Agency, 2018). At present, the implementation of FPNs is not a specific strategy to enhance food safety measures in food businesses including dark kitchens. It is also crucial to understand that such implementations will impose additional burdens on food businesses and local authorities when resources are already stretched. EHOs require clearer standards and better support and a concentrated effort to enhance clarity around regulations of dark kitchens can help to standardise enforcement of food safety standards across local authorities.

In addition to the lack of guidance, EHOs mentioned experiencing various issues relating to food hygiene and safety standards. In particular, EHOs reported the challenging working conditions associated with dark kitchens. These concerns have been echoed in previous research by Davies (2021) and Giousmpasoglou et al. (2023), who explore how the inherent nature of dark kitchens, where cost efficiency is prioritised, will create suboptimal working conditions. Resource constraints are a common issue across the food industry (Gray and Barford, 2018; Whitworth, 2024). In the case of dark kitchens, the challenge is further exacerbated by the high number of operations and irregular operating hours (Rinaldi et al., 2022; Hakim et al., 2023; Giousmpasoglou et al., 2023; da Cunha et al., 2024). Addressing these issues requires increased funding, which perhaps is not as easy to secure due to availability of resources and political will (Plume, 2018). This suggests that more innovative solutions are needed. For dark kitchens in particular, collaborative efforts between regulatory bodies, local authorities and dark kitchen operators are a cost effective and vital means to bridge the resource gap (Meyer et al., 2017).

In contrast, dark kitchen owners and tenants faced different challenges in relation to the implementation of food safety practices. The dark kitchen environment is one of convenience, flexibility and there is a potential for increased revenue (Khan, 2020; Ghazanfar et al., 2023). Often dark kitchen operators will share spaces and resources with other tenants to further reduce operating costs. While this can prove advantageous, this can lead to increased cross-contamination risks and poor food safety practices, especially where conflicts in responsibility may arise. EHOs mentioned how open communication with dark kitchen operators can help to foster better food hygiene and food safety practices. Existing research emphasised the importance of clear communication in ensuring compliance with food safety standards and how simple misunderstandings can lead to more significant issues like food-borne illness (Meyer et al., 2017; da Cunha 2021). Yapp and Fairman (2006) indicated that food businesses had often relied on EHOs for advice on compliance. Likewise, establishing a partnership with trading standards officers can reduce the resource burden for EHOs through joint inspections. Although food safety concerns were identified as a major issue by EHOs and some dark kitchen operators, however such food safety issues are not unique to dark kitchens, and they may not be necessarily worse than other types of food business operators such as takeaways. The challenge is that the dark kitchen operational model poses different issues such as shared spaces and conflicts over responsibility between different dark kitchens operating within the same premises. These factors complicate food safety implementation and inspection due to the lack of individual dark kitchens’ accountability for hygiene practices and compliance.

Furthermore, the food handling practices of delivery drivers was also highlighted as an issue by dark kitchen owners and tenants. Rarely has this aspect been considered in discussions surrounding dark kitchens, where the focus of food safety is typically on the dark kitchen operators themselves. Dark kitchen operators further mentioned how issues with untrained staff can compromise the food safety standards of their operations. This is unsurprising as in comparison to traditional restaurants, the dark kitchen operational model has a high staff turnover rate due to reduced wages, long working hours and poor working conditions (Giousmpasoglou et al., 2023). In fact, for many the concept of dark kitchens which is known to offer convenience, flexibility and low start-up costs, prioritises short term gains which is likely to attract a more transient workforce. Moreover, the constant practice of onboarding staff will strain resources due to the need for continuous training which can impact long-term success. Training for both dark kitchen operators and delivery drivers which is targeted and focuses on enhancing food safety methods would prove beneficial.

The final challenge mentioned by dark kitchen owners and tenants was the reliance on online aggregators, specifically for updating and sharing food allergen and menu changes. Previous literature has noted how the dependence on online aggregators is a recognised issue for dark kitchen operators due to their increased costs associated with onboarding (Ghazanfar et al., 2023). Additionally, there is existing research which acknowledges the responsibility of online aggregators in ensuring that food ordered through their platform is delivered in line with food safety standards (Foods Standards Agency, 2022). Online aggregators act as intermediaries between dark kitchen operators and consumers (Farah et al., 2021). Their role in providing consumers with timely up to date information about their food and hence improving food safety is crucial. The online aggregators make it possible for many of the businesses to exist and therefore need to be proactive in firstly, monitoring and validating food businesses that use their platformsOnline aggregators should also be more proactive in firstly, monitoring and validating food businesses that use their platforms and secondly, in sharing vital information with local authorities and EHOs. In their report, the FSA (2021) highlight how major platforms have the ability to ensure rigorous vetting of food businesses and ongoing monitoring. Therefore, online aggregators can help in mitigating food safety risks and collaboration with such aggregators is key. For example, new FBOs wishing to join Just Eat must be registered with the local authority and have a food hygiene rating of 3 (generally satisfactory), a pass in Scotland or be awaiting inspection (Whitworth, 2021).

## 5 Limitations

This study included a small sample of dark kitchen owners or tenants, likely those who were more invested in food safety standards. Those with poor food hygiene practices may have been less inclined to participate. This introduces selection bias and non-response bias from operators who might have opted out. Additionally, only two focus group discussions were conducted with EHOs which may limit the depth of insights and experiences captured regarding the identification and inspection of food safety in dark kitchens. Different Local Authorities and EHOs have varying levels of experience in dealing with dark kitchens. However, this limitation was mitigated by the mixed method approach that combined both an online survey and the focus group discussions, thus enhancing the reliability and validity of the findings. The above limitations highlight the need for more comprehensive research to address potential compliance gaps among dark kitchen operators and to ensure consistent food safety standards across this growing sector of the food industry.

## 6 Practical Implications

Challenges do exist with the dark kitchen model in relation to their identification, inspection and current food safety standards. This study indicates that more needs to be done to ensure that the inevitable growth of dark kitchens are accompanied by effective food safety measures. Although this study specifically looked at dark kitchens, the findings do not mean that dark kitchens’ food safety standards are lower than other food businesses. The operating model of dark kitchens pose different challenges due to shared kitchen spaces, multiple trading names and sporadic operational times. Our study proposed the following recommendations:

i) Provide information such as whether they are known by other trading names during registration.
ii) Provide comprehensive and specific guidance tailored to the unique operational structure of dark kitchens such as guidance around multiple, shared units in the same premises or different operating times.
iii) Provision of support for LAs and dark kitchens through increasing number of EHOs, resources and training of dark kitchens.
iv) Engage with online aggregators for information sharing and verification.
v) Engage with delivery drivers and/or third-party delivery company to ensure food safety practices.

These recommendations provide a unique perspective based on the lived experience of EHOs and dark kitchens and emphasise that gaps do exist in the current state of food safety practices of dark kitchens. Implementing such recommendations can offer more control to this new but rapidly evolving sector of the food industry. Future studies could explore the use of case comparisons to evaluate the effectiveness of the proposed recommendations for improving food safety in dark kitchens. Furthermore, future research would also benefit from investigating examples of successful dark kitchens that have demonstrated high food safety standards as these cases could provide best practices for improving compliance across the sector.

## Supplementary Material

Demographic characteristics of dark kitchen participants (n=16)

Focus group discussion topic guide (Environmental Health Officers)

Online Survey for Environmental Health Officers

Semi-structured Interview Guide – Dark kitchens

## Figures and Tables

**Table 1:** Inspecting dark kitchens (n=123)

Questions	Frequencies n (%)	
	Yes	No
Have you inspected a dark kitchen?	94 (76.4)	29 (23.6)
Do you face challenges in identifying dark kitchens?	97 (78.9)	26 (21.1)
Do you rely on dark kitchens registering their food businesses in order to identify them?	96 (78.0)	27 (22.0)
Do you pro-actively look for unregistered dark kitchens in your local authority?	43 (35.0)	80 (65.0)

**Table 2:** Challenges faced by LAs to identify dark kitchens (n=123)

Indicators/Factors	Agreement %	Factor loading
**Factor 1 – Dark kitchen invisibility**		
Dark kitchens operating under several brand names meaning the same business is operating under different names	77.2	0.765
Dark kitchens that operate as a virtual business, but their kitchen is based at a standard restaurant	47.2	0.679
The ability of dark kitchens to close and re-open their businesses at different sites	61.0	0.676
**Factor 2 – Lack of staff and funding**		
Lack of staff to proactively look for them	65.9	0.844
Lack of funding to hire more staff	45.5	0.846
**Factor 3 – Difficulties about registrations and understanding**		
Dark kitchen operators unaware of their obligation to register	64.2	0.777
Difficulty identifying location of unregistered dark kitchens	69.9	0.637
EHOs facing lack of understanding about dark kitchens	14.6	0.596

**Table 3:** Challenges faced by the LAs when inspecting a dark kitchen (n=123)

Indicators/Factors	Agreement %	Factor loading
**Factor 1 – Shared spaces**		
Several different food businesses sharing the same kitchen space but operates at different times (increases the number of visits to the same premises)	60.2	0.692
Uncertain or sporadic operating hours which makes unannounced inspections difficult	67.5	0.770
Several different food businesses sharing the same kitchen space at the same time (this makes it difficult to identify responsibility in ensuring food safety)	60.2	0.603
The same food business with different brand names using the same kitchen space	60.2	0.664
**Factor 2 - Other indicators**		
Allocating a food hygiene rating to the dark kitchens	26.8	0.779
Dark kitchens that purchased from other food businesses and sells the food	35.0	0.717

## Highlights

- Identifying un-registered dark kitchens are hindered by multiple challenges.
- Online aggregators play a major role in ensuring food safety in dark kitchens.
- Open communications are required between local authorities, dark kitchens and online aggregators.
- Delivery drivers are often overlooked but they play a crucial role in ensuring food safety.
