# Supplementary material for: ‘Going dark’ or under the radar? Challenges and opportunities for local authorities and dark kitchens in ensuring food safety
Source: Food Control. Author manuscript; Available in PMC 2025 Jun 1. (PMC7617420; doi:10.1016/j.foodcont.2025.111179)
Supplement: Demographic characteristics of dark kitchen participants (n=16) [file EMS203382-supplement-Demographic_characteristics_of_dark_kitchen_participants__n_16_.docx]

Supplementary Material 4. Demographic characteristics of dark kitchen participants (n=16)

| Participant Number | Location | Tenant/Owner |
| --- | --- | --- |
| Dark Kitchen 1 | Multiple DKs – Manchester, London | Female (Rented DK) |
| Dark Kitchen 2 | London | Male (Rented DK) |
| Dark Kitchen 3 | London | Female (Rented DK) |
| Dark Kitchen 4 | Birmingham | Female (Shared DK) |
| Dark Kitchen 5 | London | Male (Shared DK) |
| Dark Kitchen 6 | Southampton | Female (Rented DK) |
| Dark Kitchen 7 | Plymouth | Female (Rented DK) |
| Dark Kitchen 8 | Multiple DK’s - Birmingham, Manchester, London | Female (Multiple DK’s – Rented, Owned and Shared) |
| Dark Kitchen 9 | Bristol | Female (Shared DK) |
| Dark Kitchen 10 | Leicester | Male (Shared DK) |
| Dark Kitchen 11 | Portsmouth | Male (Shared DK) |
| Dark Kitchen 12 | Manchester | Male (Owner of DK) |
| Dark Kitchen 13 | London | Male (Owner of DK) |
| Dark Kitchen 14 | Liverpool | Male (Home Based DK) |
| Dark Kitchen 15 | London | Male (Rented DK) |
| Dark Kitchen 16 | Peterborough | Male (Home Based DK - *previous experience of sharing a DK*) |
