# Supplementary material for: ‘Going dark’ or under the radar? Challenges and opportunities for local authorities and dark kitchens in ensuring food safety
Source: Food Control. Author manuscript; Available in PMC 2025 Jun 1. (PMC7617420; doi:10.1016/j.foodcont.2025.111179)
Supplement: Focus group discussion topic guide (Environmental Health Officers) [file EMS203382-supplement-Focus_group_discussion_topic_guide__Environmental_Health_Officers_.docx]

**‘Going dark’ or under the radar? Challenges and opportunities for local authorities and dark kitchens in ensuring food safety for the public**

Purpose and Guideline:

We are here today to explore the challenges and opportunities for local authorities in ensuring food safety in dark kitchens. **We define dark kitchens as food services without front-facing service or direct contact with customers and offer meals transported by delivery. Home-based, rented or shared premises will be included in this context.** There are no right or wrong answers. You can agree or disagree with each other and you may change your mind at any time during the session. This is an open space and I would like you to feel comfortable speaking your mind and sharing your views. You will not be judged on what you say. Your participation is voluntary and if at any point you feel uncomfortable, you may leave.

Please feel free to switch off your camera if you like. Please speak one at a time and speak clearly. Everything you say will of course be confidential. You will not be able to be identified from what you say during this discussion. For the sake of analysis, each participant will be assigned a code (e.g., Environmental Health Officer 1) to ensure anonymity. However, to illustrate certain points, direct quotes will be included within the publication. Before we begin does anyone have any questions?

**Focus Group Discussion Topic Guide (Environmental Health Officers)**

1. Could you share your experiences or challenges when inspecting a dark kitchen?

(*Probe: Same dark kitchen operating with different brand names; Several different food businesses sharing the same kitchen space; Sporadic operating hours; Have there been any particular food safety issues in shared kitchens - any allergen or cross-contamination risks?)*)

1. How do you identify dark kitchens?
2. What about dark kitchens that are not registered? How do you identify them?

*(Probe: Did you identify unregistered dark kitchens through customers’ complaints / neighbourhood complaints?)*

1. How could we improve the food hygiene inspections of dark kitchens? (*Probe: In particular for shared kitchens – less control so more frequent inspections may be needed*)
2. How do you work with Trading Standards Officers when it comes to allergen control?
3. Do you have knowledge of any enforcement action being taken for dark kitchens failing to meet food safety standards?
4. Have you had any training / support for conducting food safety inspections in dark kitchens (*Probe: is there any sharing/learning with other EHO’s through any informal networks – Facebook/WhatsApp*).
5. Do you have any other comments?

We will be conducting a 90 minute online workshop to share and discuss the project findings in August. Could we invite you to the workshop?

Thank you for your time and input.
