# Supplementary material for: ‘Going dark’ or under the radar? Challenges and opportunities for local authorities and dark kitchens in ensuring food safety
Source: Food Control. Author manuscript; Available in PMC 2025 Jun 1. (PMC7617420; doi:10.1016/j.foodcont.2025.111179)
Supplement: Online Survey for Environmental Health Officers [file EMS203382-supplement-Online_Survey_for_Environmental_Health_Officers.docx]

**Online Survey for Environmental Health Officers**

Section I: Demographics

| No. | Question | Items |
| --- | --- | --- |
| 1 | Which Local Authority are you based at? Please specify in the textbox below. |  |
| 2 | How many years have you been working as an Environmental Health Officer? | 1 – 5 years |
|  |  | 6 – 10 years |
|  |  | 10 – 15 years |
|  |  | More than 15 years |

**Section II: Dark Kitchens**

Dark kitchens are food services without front-facing service or direct contact with customers and offer meals purchased by online delivery. Home-based, rented or shared premises will be included in this context.

| No. | Questions | Items |
| --- | --- | --- |
| 1 | Have you inspected a dark kitchen? | Yes |
|  |  | No |
|  |  |  |
| 2 | Have your Local Authority received complaints about local dark kitchens? | Yes |
|  |  | No |
|  |  | Unsure |
|  |  |  |
| 3 | If yes, could you briefly describe the complaints please? |  |
|  |  |  |
| 4 | Do you face challenges in identifying dark kitchens? | Yes |
|  |  | No |
|  |  |  |
|  | Do you pro-actively look for unregistered dark kitchens in your Local Authority? | Yes |
|  |  | No |
|  |  |  |
| 5 | How does your Local Authority identify dark kitchens that are not registered with the local authority? Please select all that apply. | We identify them based on: |
|  |  | Complaints from the neighbourhood |
|  |  | Complaints from consumers who purchased from the dark kitchens |
|  |  | Complaints from staff who worked at the dark kitchens |
|  |  | Tip-offs from other businesses or customers |
|  |  | Searches for social media presence |
|  |  | Change of use licensing requests |
|  |  | Others |
|  |  |  |
| 8a | If you selected other, please describe |  |
|  |  |  |
|  |  |  |
| 9 | Do you rely on dark kitchens registering their food businesses in order to identify them? | Yes |
|  |  | No |
|  |  |  |
| 10 | What are the challenges faced by your Local Authority to identify dark kitchens? Please select all that apply. | EHOs facing lack of understanding about dark kitchens |
|  |  | Lack of staff to proactively look for them |
|  |  | Lack of funding to hire more staff |
|  |  | Dark kitchens operating under several brand names meaning the same business is operating under different names |
|  |  | Difficulty identifying location of unregistered dark kitchens |
|  |  | Dark kitchen operators unaware of their obligation to register |
|  |  | The ability of dark kitchens to close and re-open their businesses at different sites |
|  |  | Dark kitchens that operate as a virtual business, but their kitchen is based at a standard restaurant |
|  |  | Unregistered dark kitchens |
|  |  | Proliferation of dark kitchens |
|  |  | Others |
|  |  |  |
| 10a | If you selected Other, please specify: |  |
|  |  |  |
| 11 | What are the challenges faced by your Local Authority when inspecting a dark kitchen? Please select all that apply. | Inability to identify dark kitchens, thus we cannot inspect their food safety standards onsite |
|  |  | Several different food businesses sharing the same kitchen space at the same time (this makes it difficult to identify responsibility in ensuring food safety) |
|  |  | The same food business with different brand names using the same kitchen space |
|  |  | Several different food businesses sharing the same kitchen space but operates at different times (increases the number of visits to the same premises) |
|  |  | Uncertain or sporadic operating hours which makes unannounced inspections difficult |
|  |  | Dark kitchens that purchased from other food businesses and sells the food |
|  |  | Allocating a food hygiene rating to the dark kitchens |
|  |  | Others |
|  |  |  |
| 11a | If you selected Other, please specify: |  |
|  |  |  |
| 12 | Can you describe any examples of problems that you have experienced when inspecting dark kitchens? |  |
|  |  |  |
| 9 | Do you have any ideas or strategies to identify unregistered dark kitchens? |  |
|  |  |  |
| 10 | How could we improve the food hygiene inspections of dark kitchens? |  |
