# Supplementary material for: ‘Going dark’ or under the radar? Challenges and opportunities for local authorities and dark kitchens in ensuring food safety
Source: Food Control. Author manuscript; Available in PMC 2025 Jun 1. (PMC7617420; doi:10.1016/j.foodcont.2025.111179)
Supplement: Semi-structured Interview Guide – Dark kitchens [file EMS203382-supplement-Semi_structured_Interview_Guide___Dark_kitchens.docx]

**‘Going dark’ or under the radar? Challenges and opportunities for local authorities and dark kitchens in ensuring food safety for the public**

*Purpose and Guideline:*

We are here today to explore the challenges and opportunities for dark kitchens in ensuring food safety. There are no right or wrong answers. This is an open space and I would like you to feel comfortable speaking your mind and sharing your views. You will not be judged on what you say. Your participation is voluntary and if at any point you feel uncomfortable, you may leave. Everything you say will of course be confidential. You will not be able to be identified from what you say during this discussion. For the sake of analysis, you will be assigned a code (e.g., Dark Kitchen Owner 1) to ensure anonymity. However, to illustrate certain points, direct quotes will be included within the publication. Please note that the session will be recorded, please feel free to switch off your video if you like. Before we begin do you have any questions?

**Semi-structured Interview Guide – Dark kitchens**

1. Could you tell us briefly about your food business please?
2. Could you tell us about your dark kitchen operating model please? (*Probe: For example, do you own, rent or share your dark kitchen? Why have you chosen to use this dark kitchen model? Do you find it easier to operate?*)
3. How would you define a dark kitchen?

(Probe: *What do you understand about dark kitchens?; Would you prefer a different terminology?)*

1. Which online delivery platform advertises your food business?
2. Can you tell me about the procedure for starting or renting a dark kitchen space?
3. How do you ensure the food safety of your dark kitchens?
4. How quickly does new food or food allergen information (any changes in ingredients or recipe) get updated in online delivery services? (Probe: Is there any lag time between dark kitchens informing online delivery services and them being updated? Has this or can this cause any issues?)
5. Do you offer food safety training and support for your dark kitchen tenants? (*Probe: Does this include any specific food safety training for food allergies and to reduce cross contamination?)*
6. Have there been any challenges for food safety inspections of dark kitchens? (*Probe: Have there been any issues in maintaining food safety (food allergens/cross contamination) in shared kitchens?).*
7. How do you think we can improve the current food safety inspections of dark kitchens?
8. What are the challenges of operating a dark kitchen?

*(Probe: Lack of staff; High staff turnover; Competition)*

We will be conducting a 60 to 90-minute online workshop to share and discuss the project findings in August. Could we invite you to the workshop please?

Thank you for your time and input.
